# Endovascular Thrombectomy in Patients With Cancer and Acute Ischemic Stroke: A Systematic Review and Meta-Analysis

**DOI:** 10.7759/cureus.116605

**Published:** 2026-09-20

**Authors:** Nooruldeen H Ali Al-Khafaji, Ali K Al-Shalchy

**Affiliations:** 1 Department of Surgery, College of Medicine, University of Baghdad, Baghdad, IRQ

**Keywords:** acute ischemic stroke (ais), cancer-associated stroke, endovascular thrombectomy, functional outcome, malignancy

## Abstract

Patients with cancer and acute ischemic stroke are underrepresented in randomized thrombectomy trials, and the safety and effectiveness of endovascular thrombectomy (EVT) in this population remain uncertain. We systematically reviewed clinical, procedural, and safety outcomes of EVT in cancer patients compared to non-cancer controls. Searches of PubMed, Scopus, and the Cochrane Library identified 10 observational studies. Meta-analyses summarized outcomes within cancer groups, while comparative analyses calculated risk differences (RDs) as cancer minus control. Bias was assessed using the ROBINS-I (Risk of Bias In Non-randomized Studies of Interventions) tool. Among cancer patients undergoing EVT, the pooled success rate for recanalization was 77.1% (95% CI, 71.2%-83.0%). Symptomatic intracranial hemorrhage (sICH) occurred in 4.5% (95% CI, 2.6%-6.5%). A favorable 90-day functional outcome (modified Rankin Scale (mRS) ≤2) was seen in 26.8% (95% CI, 20.6%-32.9%), with 39.4% (95% CI, 31.6%-47.2%) dying within 90 days. Comparative analyses showed that cancer patients were significantly less likely to attain mRS ≤2 at 90 days (RD, -0.170; 95% CI, -0.223 to -0.116; I² = 23.4%) and had higher 90-day mortality (RD, 0.234; 95% CI, 0.168 to 0.300; I² = 45.1%). No significant differences were found for successful recanalization (RD, -0.033; 95% CI, -0.083 to 0.016; I² = 31.5%) or sICH (RD, -0.001; 95% CI, -0.020 to 0.018; I² = 0%). Sensitivity analyses excluding high-bias studies produced similar results. EVT can yield high recanalization rates with low sICH risk in cancer patients; however, they show poorer functional recovery and higher 90-day mortality than controls. These results emphasize the need for personalized treatment considering both stroke and cancer prognosis.

## Introduction and background

Cancer-related stroke is an increasingly recognized subtype of acute ischemic stroke (AIS). About 10%-15% of stroke patients are reported to have malignancy, with many having active disease [1,2]. It is crucial to distinguish between active cancer, metastatic disease, and a history of malignancy, as these groups differ in prognosis, treatment, and stroke mechanisms. However, definitions of these categories vary across studies. The link between cancer and stroke is bidirectional: cancer increases the risk of ischemic stroke, and stroke may sometimes be the first sign of hidden cancer, especially within the first year after the event [3,4]. The mechanisms behind stroke in cancer patients are diverse; apart from traditional vascular causes, active cancer can contribute through systemic hypercoagulability, inflammation, nonbacterial thrombotic endocarditis, and tumor-related prothrombotic pathways [2,5]. Therefore, cancer-associated stroke often occurs in the context of systemic disease and poses unique diagnostic and treatment challenges.

Endovascular thrombectomy (EVT) is a proven treatment for suitable patients with AIS caused by large-vessel occlusion. However, its application and expected results in cancer patients are less clear, as this group has been underrepresented in randomized thrombectomy trials [1]. This may be due to restrictive trial criteria, limited life expectancy, advanced systemic disease, bleeding risks, and treatment-related comorbidities common in some malignancy patients. Consequently, much of the evidence comes from observational cohorts and administrative data. Treatment choices are further complicated by factors such as cancer activity, metastatic burden, systemic disease severity, pre-stroke functional status, and overall prognosis, all of which can independently affect outcomes regardless of thrombectomy success [1,2,5]. This systematic review and meta-analysis examined the results of EVT in adults with cancer and AIS. The main focus was on functional independence at 90 days, with secondary outcomes including mortality, successful recanalization, and symptomatic intracranial hemorrhage (sICH). Besides summarizing the outcomes within cancer groups, we also compared these results directly with those from non-cancer EVT control groups.

## Review

Materials and methods

Study Design and Search Strategy

This systematic review and meta-analysis followed the Preferred Reporting Items for Systematic Reviews and Meta-Analyses (PRISMA) 2020 guidelines [6]. Searches were conducted in PubMed, Scopus, and the Cochrane Library from their inception until February 18, 2026. The PubMed search used: ("Mechanical Thrombectomy"[Mesh] OR "mechanical thrombectomy" OR "endovascular thrombectomy") AND ("Stroke"[Mesh] OR "acute ischemic stroke") AND ("Neoplasms"[Mesh] OR cancer OR malignancy OR tumor). The strategies for Scopus and Cochrane Library were: (mechanical thrombectomy OR endovascular thrombectomy) AND (acute ischemic stroke OR ischemic stroke) AND (cancer OR malignancy OR neoplasm OR tumor). Additional eligible studies were identified by screening reference lists of included studies and relevant systematic reviews. The review protocol was not prospectively registered.

Eligibility Criteria

Included studies comprised observational cohort studies, registry-based studies, and administrative database research involving adults with AIS treated via EVT. To be eligible, studies needed to report a cancer cohort undergoing EVT with extractable data for at least one outcome of interest. Additionally, studies featuring a non-cancer EVT comparator were suitable for the comparative meta-analyses. Cancer status was determined based on each original study's criteria and could encompass active, metastatic, remote, previous, or unspecified cancer diagnoses. Due to variations in how cancer status was defined across studies, the cancer populations were not presumed to be clinically uniform. Studies needed to provide extractable data on at least one key outcome, such as successful recanalization, functional results, mortality, or hemorrhagic complications. Exclusions included case reports, small case series, non-human studies, studies focused solely on intravenous thrombolysis without an EVT-treated group, and those lacking EVT-specific outcome data. When potential overlaps in populations were found, the most comprehensive or latest dataset was kept to avoid including duplicate patients. Two reviewers independently screened titles and abstracts and then evaluated potentially eligible full-text articles. Any disagreements were resolved through discussion and reaching consensus.

Data Extraction and Outcomes

Data extraction was carried out using a predefined standardized form. The variables collected included study characteristics, patient demographics, cancer-related variables (such as active and metastatic disease), treatment details, and clinical outcomes. Outcomes of interest encompassed successful recanalization, functional independence (defined as a modified Rankin Scale (mRS) score ≤2 at 90 days when available [7]), mortality at clinically defined time points, including in-hospital and 90-day mortality, and intracranial hemorrhage (ICH), including sICH as specified by individual studies. For each outcome, the numerator and the corresponding outcome-specific denominator were extracted. If follow-up or outcome ascertainment was incomplete, the denominator reported for that specific outcome was used instead of the overall study sample size.

Statement of Human and Animal Rights

This study is a systematic review and meta-analysis of previously published studies and did not involve direct contact with human participants, animal subjects, or the collection of new patient-level data. Therefore, institutional review board approval and informed consent were not required. All included studies were expected to have been conducted in accordance with the ethical standards of their respective institutions and with the principles of the Declaration of Helsinki.

Risk of Bias Assessment

The risk of bias in the included non-randomized studies was assessed using the ROBINS-I (Risk of Bias In Non-randomized Studies of Interventions) tool [8]. Each study was evaluated across several domains, including confounding, participant selection, intervention classification, deviations from intended interventions, missing data, measurement of outcomes, and selection of reported results. Studies were then categorized into low, moderate, serious, or critical risk of bias categories (Table 1) [9-18].

**Table 1:** ROBINS risk of bias assessment. ROBINS, Risk of Bias In Non-randomized Studies of Interventions.

Study	Confounding	Selection of participants	Classification of interventions/exposure	Deviations from intended interventions	Missing data	Measurement of outcomes	Selection of reported results	Overall
Verschoof et al., 2022 [9]	Moderate	Low	Low	Low	Low	Low	Moderate	Moderate
Lee et al., 2021 [10]	Serious	Moderate	Moderate	Low	Moderate	Low	Moderate	Serious
Włodarczyk et al., 2024 [11]	Moderate	Low	Low	Low	Low	Low	Moderate	Moderate
Hsieh et al., 2025 [12]	Moderate	Low	Moderate	Low	Moderate	Low	Moderate	Moderate
Shapiro et al., 2022 [13]	Moderate	Low	Moderate	Low	Low	Moderate	Moderate	Moderate
Ciolli et al., 2021 [14]	Serious	Moderate	Low	Low	Moderate	Moderate	Moderate	Serious
Terazono et al., 2024 [15]	Serious	Moderate	Moderate	Low	Moderate	Moderate	Moderate	Serious
Merlino et al., 2021 [16]	Moderate	Moderate	Moderate	Low	Moderate	Low	Moderate	Moderate
Rinaldo et al., 2019 [17]	Serious	Low	Moderate	Low	Low	Moderate	Moderate	Serious
Fu et al., 2024 [18]	Serious	Serious	Low	Low	Moderate	Low	Moderate	Serious

Statistical Analysis

Single-arm meta-analyses estimated pooled proportions among cancer patients undergoing EVT. Only studies with both an event count and a relevant denominator for each outcome were included. When the number of patients with available outcome data varied from the total study population, outcome-specific denominators were used. Missing or unavailable outcomes were considered missing and not recorded as zero. Pooled proportions and 95% confidence intervals (CIs) were derived using random-effects models.

We conducted comparative meta-analyses for four predefined outcomes: functional independence at 90 days (mRS ≤2), 90-day mortality, successful recanalization, and sICH. Study-specific effects were expressed as absolute risk differences (RDs), calculated by subtracting the event proportion in the non-cancer group from that in the cancer group. A negative RD indicates a lower event rate in patients with cancer, while a positive RD shows a higher event rate. These analyses were based on raw event counts and outcome-specific denominators for both the cancer and non-cancer EVT groups. Random-effects models combined study-specific RDs with 95% CIs. Heterogeneity among studies was evaluated using the I² statistic and τ² (between-study variance). For the main random-effects meta-analyses, 95% prediction intervals (PIs) were also calculated to describe the possible range of true effects or proportions in future similar studies. These PIs were interpreted with caution due to the limited number of studies in each analysis. Missing outcome data were considered unavailable and were excluded from the respective outcome analysis, resulting in a variable number of studies contributing to each outcome.

Sensitivity analyses involved re-running each meta-analysis after removing studies classified as having a serious overall risk of bias based on ROBINS-I. Sensitivity analyses were not performed when fewer than two studies with usable outcome data remained after these exclusions. These sensitivity estimates were then compared to the primary analyses to evaluate the stability of the pooled results. A formal assessment of publication bias was not performed for meta-analyses with fewer than 10 studies, as funnel-plot asymmetry tests tend to have low power and can produce unreliable results with a limited number of studies. All analyses were carried out using jamovi version 2.7.12.0 (The Jamovi Project, Sydney, Australia) with the esci (Estimation Statistics with Confidence Intervals) meta-analysis module. The primary basis for statistical inference was the pooled effect estimates along with their 95% CIs.

Results

Study and Patient Characteristics

A total of 664 records were identified, with 577 remaining after removing duplicates. After screening and full-text review, 10 studies were included in the systematic review (Figure 1). Overall, the comparative studies included were assessed to have a moderate to serious risk of bias. Key issues included residual confounding typical of observational studies, incomplete adjustment for cancer severity and prestroke conditions, varied cancer definitions, reliance on administrative coding in large database research, small sample sizes for cancer groups in single-center cohorts, and selective inclusion of EVT patients with retrieved clots in one study.

**Figure 1:**
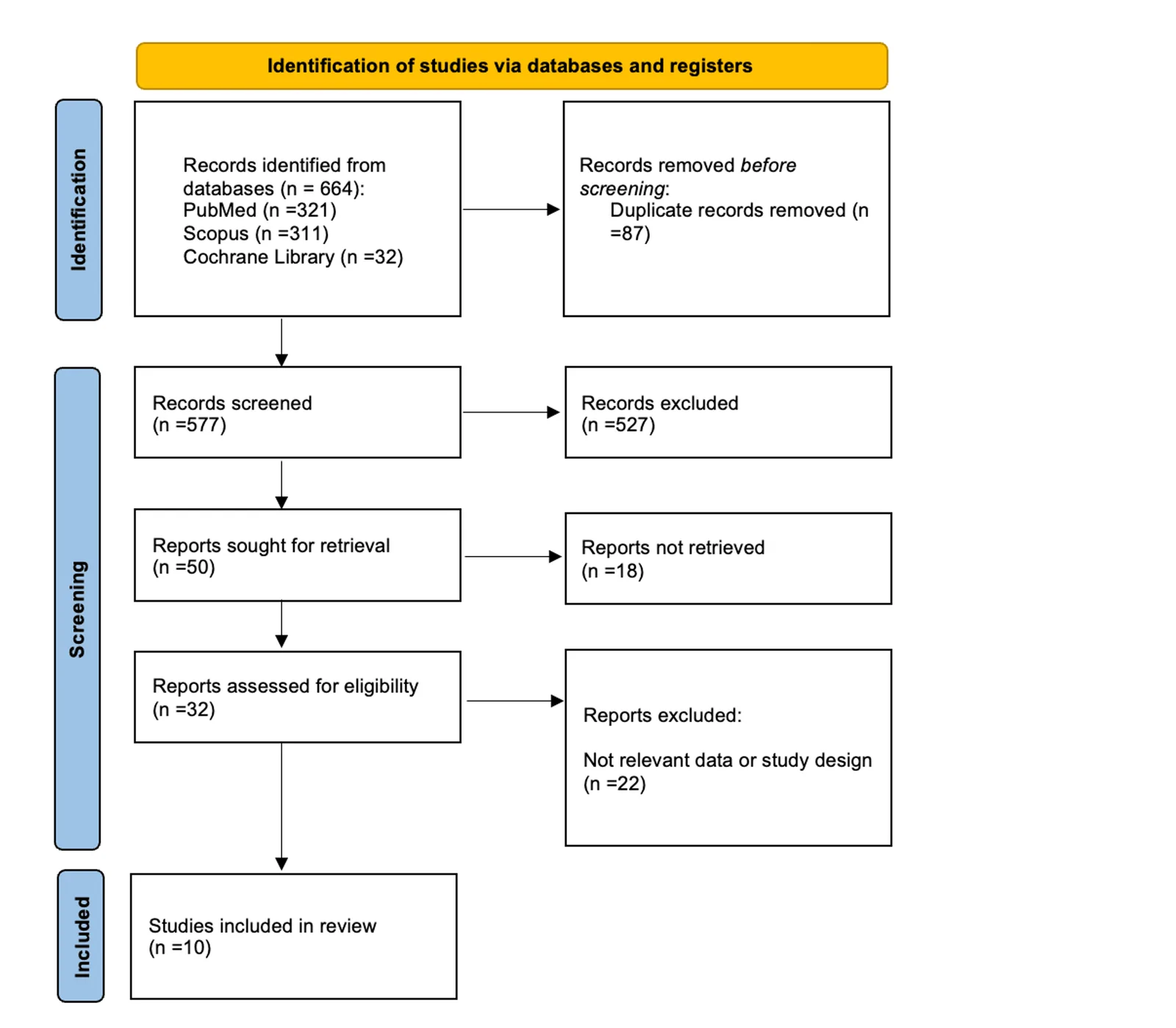
PRISMA flow diagram. Eighteen reports could not be retrieved because full-text articles were not accessible through available online or institutional resources and could not be obtained by other means at the time of the review. PRISMA, Preferred Reporting Items for Systematic Reviews and Meta-Analyses.

The number of studies included in each single-arm meta-analysis depended on outcome availability. The combined proportion of male patients was 50.1% (95% CI, 49.2%-51.1%; 95% PI, 49.2%-51.1%; I² = 0%; τ² = 0; k = 9). Metastatic cancer was observed in a pooled estimate of 42.8% (95% CI, 19.4%-66.2%; 95% PI, −13.0% to 98.6%; I² = 96.9%; τ² = 0.0668; k = 5), indicating substantial heterogeneity across studies. Prestroke functional independence, defined as mRS ≤2, was observed in 83.1% of patients (95% CI, 74.5%-91.8%; 95% PI, 70.2%-96.1%; I² = 62.1%; τ² = 0.00241; k = 2). Conversely, 16.9% had a prestroke mRS >2 (95% CI, 8.2%-25.5%; 95% PI, 4.0%-29.8%; I² = 62.1%; τ² = 0.00241; k = 2). Due to only two studies informing these prestroke functional-status estimates, caution is advised when interpreting these results (Tables 2-4) [9-18].

**Table 2:** Pooled single-arm estimates among patients with cancer undergoing EVT. Note: Prediction intervals were calculated from the random-effects model. For highly heterogeneous proportion estimates, prediction limits may extend beyond the natural 0%-100% bounds and should be interpreted as reflecting model uncertainty rather than clinically possible proportions. CI, confidence interval; EVT, endovascular thrombectomy; ICH, intracranial hemorrhage; IV, intravenous; mRS, modified Rankin Scale; PI, prediction interval; τ², between-study variance.

Outcome	Studies (k)	Pooled proportion, %	95% CI, %	95% PI, %	I², %	τ²
Patient/treatment characteristics						
Male sex	9	50.1	49.2–51.1	49.2–51.1	0.0	0
IV thrombolysis	9	30.1	17.3–42.9	−8.2–68.4	96.5	0.0340
Metastatic cancer	5	42.8	19.4–66.2	−13.0–98.6	96.9	0.0668
Prestroke mRS ≤2	2	83.1	74.5–91.8	70.2–96.1	62.1	0.00241
Prestroke mRS >2	2	16.9	8.2–25.5	4.0–29.8	62.1	0.00241
Procedural/safety outcomes						
Successful recanalization	7	77.1	71.2–83.0	64.7–89.6	56.5	0.00311
Any ICH	5	30.2	15.2–45.3	−3.5–64.0	88.1	0.0238
Symptomatic ICH	5	4.5	2.6–6.5	2.6–6.5	0.0	0
Functional outcomes						
mRS ≤2 at 90 days	6	26.8	20.6–32.9	14.7–38.8	53.4	0.00279
mRS >2 at 90 days	6	73.2	67.1–79.4	61.2–85.3	53.4	0.00279
Mortality outcomes						
In-hospital mortality	7	19.3	14.0–24.7	7.6–31.0	74.8	0.00282
90-day mortality	7	39.4	31.6–47.2	21.6–57.2	67.8	0.00669

**Table 3:** Study characteristics and demographics. Note: Values are presented as n/N, where n is the number of patients with the characteristic, and N is the corresponding available denominator. EVT, endovascular thrombectomy; N, number; NR, not reported.

Study	Cancer, EVT, N	Control, EVT, N	Age, cancer	Age, control	Male, cancer, n/N (%)	Male, control, n/N (%)	Metastatic cancer, n/N (%)
Verschoof et al., 2022 [9]	124	2,459	69 ± 11	70 ± 14	58/124 (46.8)	1,277/2,459 (51.9)	68/93 (73.1)
Lee et al., 2021 [10]	34	307	64.5 ± 11.4	68.9 ± 14.0	17/34 (50.0)	161/307 (52.4)	22/34 (64.7)
Włodarczyk et al., 2024 [11]	35	558	71 (IQR 14)	70 (IQR 17)	14/35 (40.0)	297/558 (53.2)	14/35 (40.0)
Hsieh et al., 2025 [12]	294	549	71.6 ± 11.8	71.4 ± 13.4	156/294 (53.1)	301/549 (54.8)	NR
Shapiro et al., 2022 [13]	8,935	123,275	69.79 ± 12.89	68.87 ± 14.40	4,465/8,935 (50.0)	64,750/123,275 (52.5)	2,490/8,935 (27.9)
Ciolli et al., 2021 [14]	14	267	73 (61–78)	72 (60–79)	8/14 (57.1)	135/267 (50.6)	1/14 (7.1)
Terazono et al., 2024 [15]	16	55	71.5 ± 10.1	72.0 ± 14.4	7/16 (43.8)	29/55 (52.7)	NR
Merlino et al., 2021 [16]	29	144	NR	NR	NR	NR	NR
Rinaldo et al., 2019 [17]	857	16,411	NR	NR	449/857 (52.4)	8,286/16,411 (50.5)	NR
Fu et al., 2024 [18]	73	347	NR	71.5 ± 12.8	32/73 (43.8)	173/347 (49.9)	NR

**Table 4:** Baseline stroke severity and prestroke functional status. Note: Values are presented as n/N, where n is the number of patients with the characteristic, and N is the outcome-specific denominator available for that study and outcome. NIHSS, National Institutes of Health Stroke Scale; IQR, interquartile range; mRS, modified Rankin Scale; NR, not reported.

Study	NIHSS, cancer	NIHSS, control	Prestroke mRS ≤2, cancer, n/N	Prestroke mRS >2, cancer, n/N	Prestroke mRS ≤2, control, n/N	Prestroke mRS >2, control, n/N
Verschoof et al., 2022 [9]	16 (12–19)	16 (11–19)	97/123	26/123	2,181/2,421	240/2,421
Lee et al., 2021 [10]	18 (11–23)	15 (8–19)	NR	NR	NR	NR
Włodarczyk et al., 2024 [11]	14 (IQR 10)	16 (IQR 10)	NR	NR	NR	NR
Hsieh et al., 2025 [12]	14.1 ± 7.1	14.2 ± 6.8	NR	NR	NR	NR
Shapiro et al., 2022 [13]	14.1 (mean)	14.1 (mean)	NR	NR	NR	NR
Ciolli et al., 2021 [14]	20 (10–23)	16 (10–21)	NR	NR	NR	NR
Terazono et al., 2024 [15]	22.6 ± 6.8	21.5 ± 10.7	NR	NR	NR	NR
Merlino et al., 2021 [16]	NR	NR	NR	NR	NR	NR
Rinaldo et al., 2019 [17]	NR	NR	NR	NR	NR	NR
Fu et al., 2024 [18]	NR	NR	64/73	9/73	322/347	25/347

Treatment Characteristics and Functional Outcomes

Intravenous thrombolysis prior to EVT was given in 30.1% of cases (95% CI, 17.3%-42.9%; 95% PI, −8.2% to 68.4%; I² = 96.5%; τ² = 0.0340; k = 9), showing substantial heterogeneity among studies. Recanalization success was reached in 77.1% of cases (95% CI, 71.2%-83.0%; 95% PI, 64.7%-89.6%), showing moderate heterogeneity (I² = 56.5%; τ² = 0.00311; k = 7). At 90 days, 26.8% of participants achieved functional independence, defined as mRS ≤2 (95% CI: 20.6%-32.9%; 95% PI: 14.7%-38.8%; I² = 53.4%; τ² = 0.00279; k = 6). In contrast, the pooled proportion of patients with an unfavorable functional outcome, defined as mRS >2, was 73.2% (95% CI: 67.1%-79.4%; 95% PI: 61.2%-85.3%; I² = 53.4%; τ² = 0.00279; k = 6) (Tables 5, 6) [9-18].

**Table 5:** Treatment characteristics. Note: Values are presented as n/N, where n is the number of patients with the event, and N is the outcome-specific denominator available for that study and outcome. IV, intravenous; EVT, endovascular thrombectomy; AF, atrial fibrillation; NR, not reported.

Study	IV thrombolysis, cancer, n/N (%)	IV thrombolysis, control, n/N (%)	AF, cancer, n/N (%)	AF, control, n/N (%)
Verschoof et al., 2022 [9]	69/123 (56.6)	1,862/2,457 (75.8)	32/122 (26.2)	582/2,434 (23.9)
Lee et al., 2021 [10]	9/34 (26.5)	80/307 (26.1)	0/34 (0.0)	175/307 (57.0)
Włodarczyk et al., 2024 [11]	9/35 (25.7)	330/558 (59.1)	13/35 (37.1)	237/558 (42.5)
Hsieh et al., 2025 [12]	68/294 (23.1)	142/549 (25.9)	119/294 (40.5)	212/549 (38.6)
Shapiro et al., 2022 [13]	695/8,935 (7.8)	14,845/123,275 (12.0)	3,020/8,935 (33.8)	46,270/123,275 (37.5)
Ciolli et al., 2021 [14]	5/14 (35.7)	127/267 (47.6)	NR	NR
Terazono et al., 2024 [15]	2/16 (12.5)	24/55 (43.6)	5/16 (31.3)	35/55 (63.6)
Merlino et al., 2021 [16]	19/29 (65.5)	95/144 (66.0)	NR	NR
Rinaldo et al., 2019 [17]	NR	NR	NR	NR
Fu et al., 2024 [18]	16/73 (21.9)	129/347 (37.2)	35/73 (47.9)	235/347 (67.7)

**Table 6:** Recanalization and functional outcomes. Note: Values are presented as n/N, where n is the number of patients with the event, and N is the outcome-specific denominator available for that study and outcome. mRS, modified Rankin Scale; 90 d, 90 days; N, number; NR, not reported.

Study	Recanalization, cancer, n/N	Recanalization, control, n/N	mRS ≤2 at 90 d, cancer, n/N	mRS ≤2 at 90 d, control, n/N	mRS >2 at 90 d, cancer, n/N	mRS >2 at 90 d, control, n/N
Verschoof et al., 2022 [9]	82/121	1,451/2,397	26/115	976/2,318	89/115	1,342/2,318
Lee et al., 2021 [10]	26/34	269/307	11/34	NR	23/34	NR
Włodarczyk et al., 2024 [11]	28/35	492/558	17/35	311/543	18/35	232/543
Hsieh et al., 2025 [12]	226/285	398/484	68/294	196/549	226/294	353/549
Shapiro et al., 2022 [13]	NR	NR	NR	NR	NR	NR
Ciolli et al., 2021 [14]	10/14	208/267	3/14	117/267	11/14	150/267
Terazono et al., 2024 [15]	NR	NR	NR	NR	NR	NR
Merlino et al., 2021 [16]	20/29	119/144	NR	NR	NR	NR
Rinaldo et al., 2019 [17]	NR	NR	NR	NR	NR	NR
Fu et al., 2024 [18]	64/73	319/347	17/72	153/316	55/72	163/316

Mortality and Safety Outcomes

In-hospital mortality was 19.3% overall (95% CI, 14.0%-24.7%; with a 95% PI of 7.6%-31.0%; I² = 74.8%; τ² = 0.00282; across seven studies). After 90 days, the pooled mortality increased to 39.4% (95% CI, 31.6%-47.2%; with a 95% PI of 21.6%-57.2%; I² = 67.8%; τ² = 0.00669; k = 7) (Table 7) [9-18].

**Table 7:** Mortality outcomes. Note: Values are presented as n/N, where n is the number of patients with the event, and N is the outcome-specific denominator available for that study and outcome. N, number; NR, not reported.

Study	In-hospital mortality, cancer, n/N	In-hospital mortality, control, n/N	90-day mortality, cancer, n/N	90-day mortality, control, n/N
Verschoof et al., 2022 [9]	29/115	353/2,318	60/115	614/2,318
Lee et al., 2021 [10]	7/34	18/307	9/34	21/307
Włodarczyk et al., 2024 [11]	7/35	74/558	12/35	121/543
Hsieh et al., 2025 [12]	56/294	47/549	104/294	89/549
Shapiro et al., 2022 [13]	1,170/8,935	12,145/123,275	NR	NR
Ciolli et al., 2021 [14]	6/14	15/267	9/14	38/267
Terazono et al., 2024 [15]	NR	NR	NR	NR
Merlino et al., 2021 [16]	4/29	NR	10/29	NR
Rinaldo et al., 2019 [17]	NR	NR	NR	NR
Fu et al., 2024 [18]	NR	NR	26/72	22/316

The pooled proportion of sICH was 4.5% (95% CI, 2.6%-6.5%; 95% PI, 2.6%-6.5%; I² = 0%; τ² = 0; k = 5). Conversely, the occurrence of any ICH was much higher at 30.2% (95% CI, 15.2%-45.3%; 95% PI, −3.5% to 64.0%; I² = 88.1%; τ² = 0.0238; k = 5), showing substantial heterogeneity (Table 8) [9-18].

**Table 8:** Hemorrhagic outcomes. Note: Values are presented as n/N, where n is the number of patients with the event, and N is the outcome-specific denominator available for that study and outcome. ICH, intracranial hemorrhage; sICH, symptomatic intracranial hemorrhage; N, number; NR, not reported.

Study	sICH, cancer, n/N	sICH, control, n/N	Any ICH, cancer, n/N	Any ICH, control, n/N
Verschoof et al., 2022 [9]	8/124	146/2,459	NR	NR
Lee et al., 2021 [10]	NR	NR	14/34	73/307
Włodarczyk et al., 2024 [11]	NR	NR	5/35	121/558
Hsieh et al., 2025 [12]	14/294	24/549	NR	NR
Shapiro et al., 2022 [13]	NR	NR	1,225/8,935	16,820/123,275
Ciolli et al., 2021 [14]	2/14	16/267	6/14	108/267
Terazono et al., 2024 [15]	NR	NR	NR	NR
Merlino et al., 2021 [16]	2/29	NR	14/29	NR
Rinaldo et al., 2019 [17]	NR	NR	NR	NR
Fu et al., 2024 [18]	1/73	9/347	NR	NR

Comparative Outcomes

A two-arm meta-analysis compared cancer patients with non-cancer controls receiving EVT, emphasizing key clinical outcomes. Random-effects meta-analyses used RDs, defined as the event proportion in the cancer group minus that in the control group. Negative values denote a lower event rate in cancer patients, while positive values indicate a higher rate. Sensitivity analyses excluded studies judged to have serious overall risk of bias using ROBINS-I (Table 9).

**Table 9:** Comparative outcomes of EVT in patients with cancer versus patients without cancer. CI, confidence interval; EVT, endovascular thrombectomy; mRS, modified Rankin Scale; PI, prediction interval; RD, risk difference; sICH, symptomatic intracranial hemorrhage.

Outcome	Studies (k)	Main RD (95% CI)	95% PI	I², %	τ²	Sensitivity (k)	Sensitivity RD (95% CI)	I², %	τ²
mRS ≤2 at 90 days	5	−0.170 (−0.223 to −0.116)	−0.248 to −0.091	23.4%	0.000864	3	−0.148 (−0.201 to −0.095)	14.2	0.000352
90-day mortality	6	0.234 (0.168 to 0.300)	0.111 to 0.357	45.1%	0.00279	3	0.204 (0.146 to 0.262)	17.8	0.000527
Successful recanalization	7	−0.033 (−0.083 to 0.016)	−0.120 to 0.053	31.5%	0.00130	4	−0.024 (−0.101 to 0.054)	56.8	0.00336
sICH	4	−0.001 (−0.020 to 0.018)	−0.020 to 0.018	0%	0	2	0.004 (−0.020 to 0.029)	0	0

Five studies offered available comparative data on favorable 90-day functional outcomes. Patients with cancer were notably less likely to reach mRS ≤2 at 90 days compared to non-cancer controls, with a pooled RD for mRS ≤2 of −0.170 (95% CI, −0.223 to −0.116; I² = 23.4%; τ² = 0.000864), indicating a 17.0 percentage point lower likelihood of independence among cancer patients (Figure 2).

**Figure 2:**
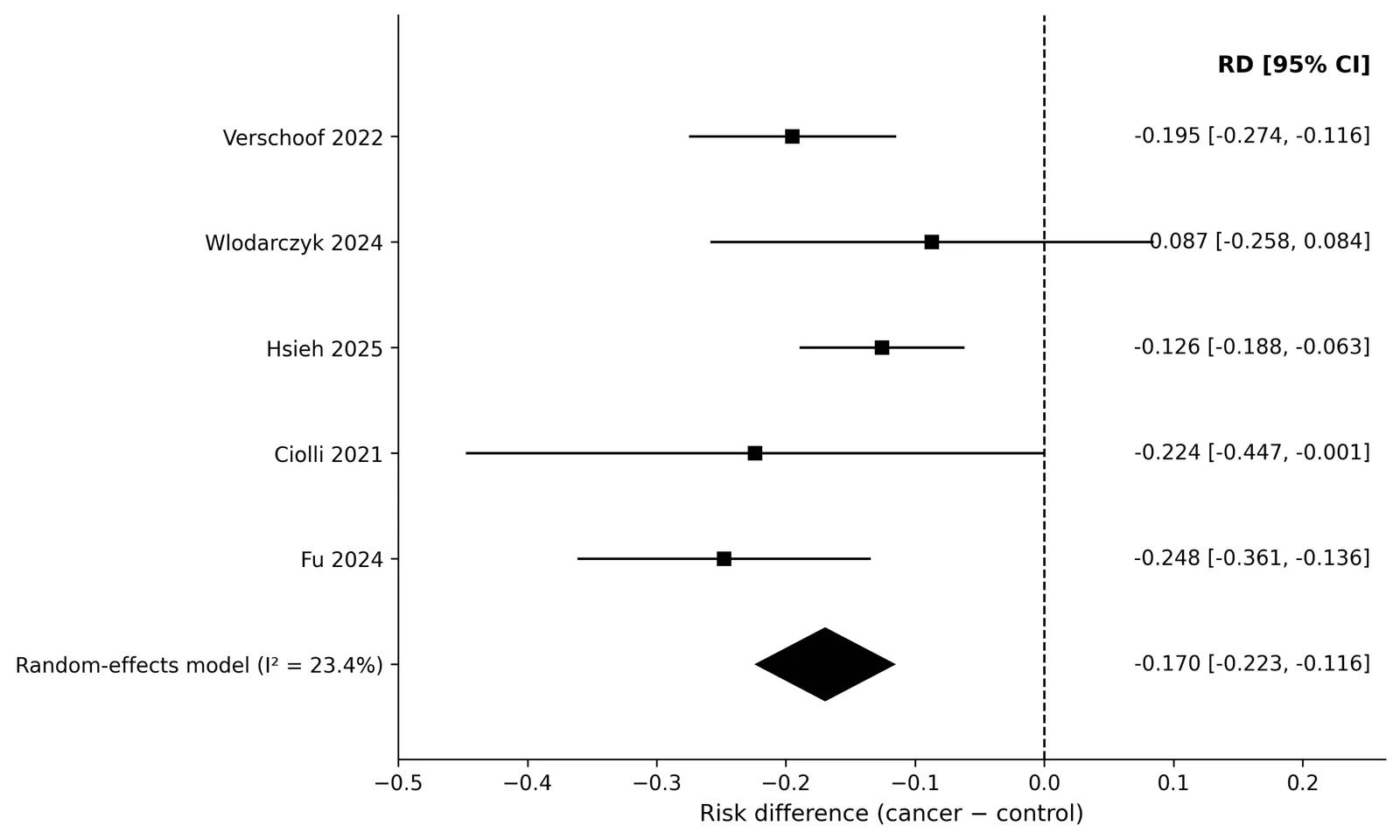
Forest plot of the risk difference in favorable functional outcome, defined as modified Rankin Scale (mRS) ≤2 at 90 days, between patients with cancer and non-cancer controls undergoing endovascular thrombectomy (EVT). The pooled risk difference (RD) was −0.170 (95% CI, −0.223 to −0.116; I² = 23.4%), indicating a lower probability of functional independence among patients with cancer. Verschoof et al., 2022 [9], Włodarczyk et al., 2024 [11], Hsieh et al., 2025 [12], Ciolli et al., 2021 [14], Fu et al., 2024 [18].

Similarly, six studies offered comparable mortality data. Cancer patients faced higher 90-day mortality rates. Across six studies, the combined RD was 0.234 (95% CI, 0.168-0.300; I² = 45.1%; τ² = 0.00279), representing a 23.4 percentage point increase in 90-day mortality (Figure 3).

**Figure 3:**
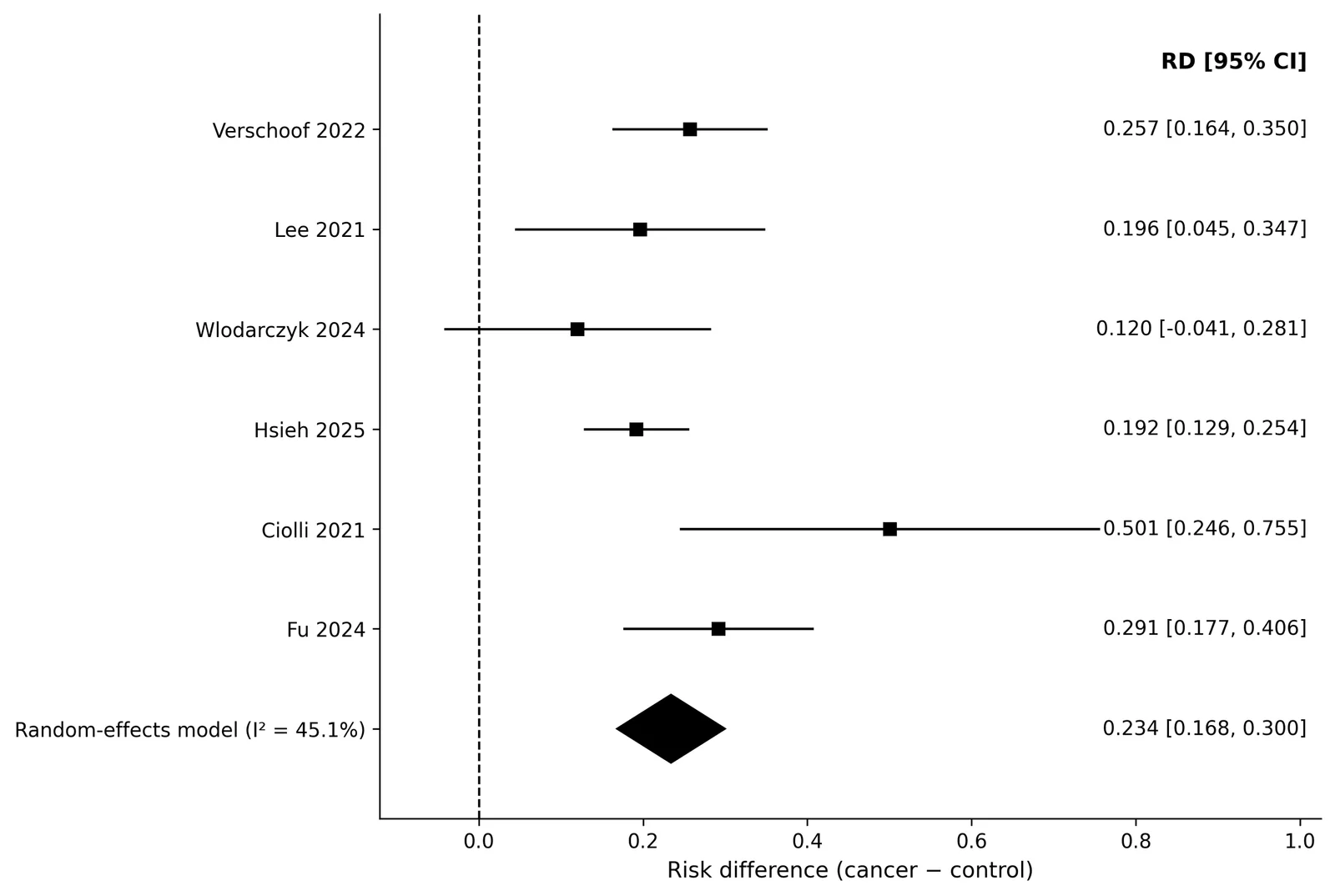
Forest plot of the risk difference in 90-day mortality between patients with cancer and non-cancer controls undergoing EVT. The pooled risk difference was 0.234 (95% CI, 0.168–0.300; I² = 45.1%), indicating higher 90-day mortality among patients with cancer. Verschoof et al., 2022 [9], Lee et al., 2021 [10], Włodarczyk et al., 2024 [11], Hsieh et al., 2025 [12], Ciolli et al., 2021 [14], Fu et al., 2024 [18]. EVT, endovascular thrombectomy; RD, risk difference.

In contrast, there was no statistically significant difference in successful recanalization rates between patients with and without cancer. The seven studies collectively showed a pooled RD of −0.033 (95% CI, −0.083 to 0.016; I² = 31.5%; τ² = 0.00130) (Figure 4).

**Figure 4:**
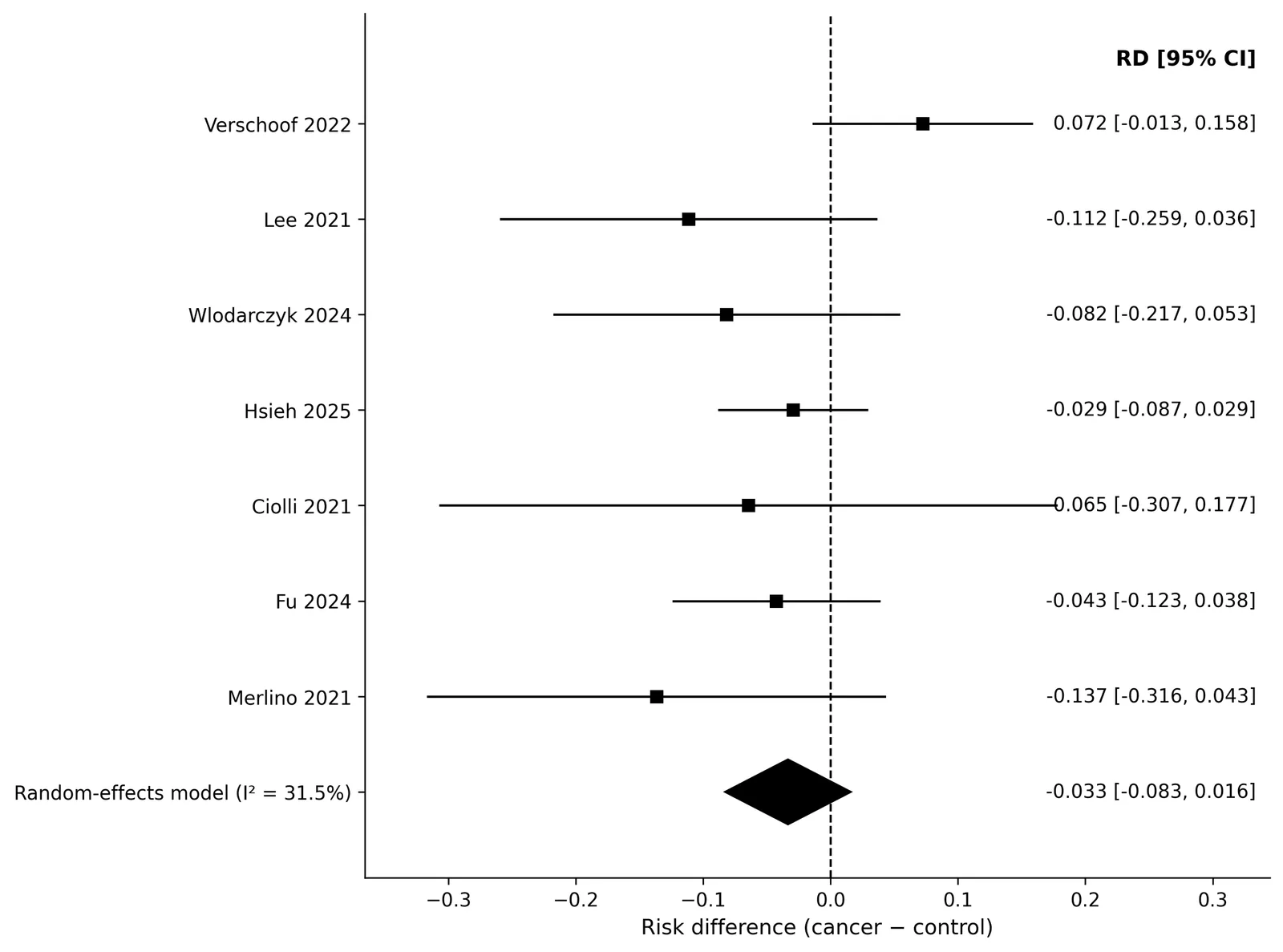
Forest plot of the risk difference in successful recanalization between patients with cancer and non-cancer controls undergoing EVT. The pooled risk difference was −0.033 (95% CI, −0.083 to 0.016; I² = 31.5%). The confidence interval crossed zero, indicating that no statistically significant difference in successful recanalization was detected between groups. Verschoof et al., 2022 [9], Lee et al., 2021 [10], Włodarczyk et al., 2024 [11], Hsieh et al., 2025 [12], Ciolli et al., 2021 [14], Fu et al., 2024 [18], Merlino et al., 2021 [16]. EVT, endovascular thrombectomy; RD, risk difference.

Similarly, the pooled RD for sICH was −0.001 (95% CI, −0.020 to 0.018; I² = 0%; τ² = 0; k = 4), indicating no significant difference in sICH rates between the groups (Figure 5).

**Figure 5:**
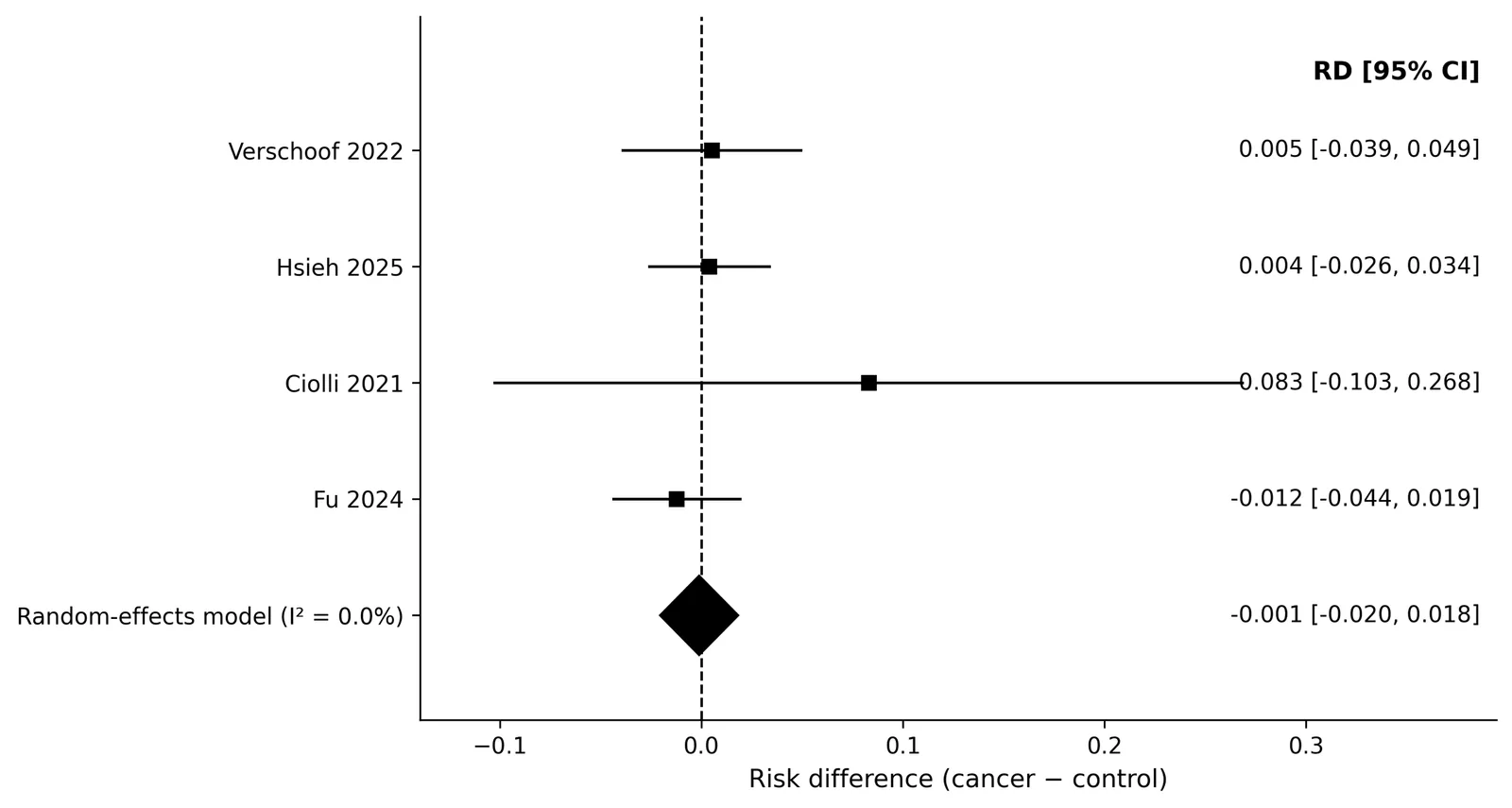
Forest plot of the risk difference in symptomatic intracranial hemorrhage (sICH) between patients with cancer and non-cancer controls undergoing EVT. The pooled risk difference was −0.001 (95% CI, −0.020 to 0.018; I² = 0%). No statistically significant difference in sICH was detected between groups. Verschoof et al., 2022 [9], Hsieh et al., 2025 [12], Ciolli et al., 2021 [14], Fu et al., 2024 [18]. EVT, endovascular thrombectomy; RD, risk difference.

The 95% PIs were −0.248 to −0.091 for functional independence, 0.111 to 0.357 for 90-day mortality, −0.120 to 0.053 for successful recanalization, and −0.020 to 0.018 for sICH. The intervals for functional independence and 90-day mortality were entirely below and above the null value, respectively, while those for successful recanalization and sICH included the null. Because each analysis was based on a small number of studies, these intervals should be interpreted with caution.

Sensitivity Analyses

Sensitivity analyses were performed after excluding studies judged to have serious overall risk of bias according to ROBINS-I. The pooled proportion of male patients was 50.0% (95% CI, 48.2%-51.8%; I² = 8.2%; τ² = 0.000075; k = 4). Intravenous thrombolysis was administered in 34.8% (95% CI, 16.3%-53.3%; I² = 98.1%; τ² = 0.0417; k = 5). Metastatic cancer was present in 46.9% (95% CI, 15.0%-78.9%; I² = 98.0%; τ² = 0.0770; k = 3). Sensitivity analyses for prestroke mRS ≤2 and prestroke mRS >2 were not conducted because only one study with appropriate data remained after removing studies at serious risk of bias.

The rate of successful recanalization was consistent at 74.7% (95% CI, 67.6%-81.7%; I² = 55.8%; τ² = 0.00271; k = 4). Regarding functional outcomes, 28.7% of patients had an mRS score of ≤2 at 90 days (95% CI, 18.1%-39.2%; I² = 79.1%; τ² = 0.00644; k = 3). Conversely, 71.3% had an mRS score >2 (95% CI, 60.8%-81.9%; I² = 79.1%; τ² = 0.00644; k = 3). The estimated sensitivity for in-hospital mortality was 17.8% (95% CI, 12.6%-23.0%; I² = 75.5%; τ² = 0.00214; k = 5), whereas the 90-day mortality rate was 39.8% (95% CI, 30.0%-49.6%; I² = 71.8%; τ² = 0.00667; k = 4). Regarding hemorrhagic outcomes, sICH was observed in 5.2% (95% CI, 3.0%-7.4%; I² = 0%; τ² = 0; k = 3), whereas any ICH was reported in 23.5% (95% CI, 7.2%-39.8%; I² = 87.3%; τ² = 0.0174; k = 3). The sensitivity estimates mostly aligned with the direction and magnitude of the primary analyses, though some outcomes still showed substantial heterogeneity.

After excluding studies with a serious overall risk of bias, the findings for functional outcomes and mortality remained consistent. For mRS ≤2 at 90 days, the pooled RD was −0.148 (95% CI, −0.201 to −0.095; I² = 14.2%; τ² = 0.000352; k = 3). The 90-day mortality sensitivity estimate was 0.204 (95% CI, 0.146-0.262; I² = 17.8%; τ² = 0.000527; k = 3).

The conclusions about procedural and safety outcomes stayed consistent. The sensitivity RD for successful recanalization was −0.024 (95% CI, −0.101 to 0.054; I² = 56.8%; τ² = 0.00336; k = 4). For sICH, the combined sensitivity RD was 0.004 (95% CI, −0.020 to 0.029; I² = 0%; τ² = 0; k = 2). Since only two studies contributed to the sICH sensitivity analysis, this estimate should be interpreted with caution.

Discussion

This systematic review and meta-analysis assessed the safety and efficacy of EVT in patients with cancer experiencing AIS. It used pooled single-arm data and direct comparisons with non-cancer controls. The key finding was a disconnect between procedural success and clinical outcomes. About 75% of cancer patients achieved successful recanalization, and sICH was rare. However, functional recovery was limited, and mortality rates remained high. In comparative analysis, cancer patients were less likely to attain functional independence at 90 days and had higher 90-day mortality, though no significant differences were found in recanalization success or sICH. Our findings should be viewed alongside earlier meta-analyses by Aloizou et al. and Duan et al. [19,20]. This review expands on previous literature by including newer comparative studies, using outcome-specific denominators, and analyzing both pooled cancer-EVT outcomes and direct cancer versus non-cancer comparisons.

The procedural success of EVT remained largely consistent in patients with cancer. The overall recanalization success rate was 77.1% (95% CI, 71.2%-83.0%), with no significant difference observed between patients with and without cancer (RD, −0.033; 95% CI, −0.083 to 0.016). The 95% PI (−0.120 to 0.053) also crossed the null, suggesting the true effect's direction may vary among similar studies. These results align with earlier reviews indicating that malignancy does not necessarily hinder successful reperfusion after EVT [19,20]. These findings are consistent with the established effectiveness of EVT for eligible patients with large-vessel occlusion in the general AIS population [21]. Likewise, the pooled sICH rate was 4.5% (95% CI, 2.6%-6.5%), with no significant difference between cancer and non-cancer groups (RD, −0.001; 95% CI, −0.020 to 0.018). The PI again included the null, and sensitivity analyses excluding studies with high bias risk yielded similar estimates for recanalization and sICH, supporting the robustness of these safety and procedural findings.

Conversely, functional recovery after EVT was considerably less favorable. Only 26.8% (95% CI, 20.6%-32.9%) of patients with cancer reached functional independence, defined as an mRS score ≤2 at 90 days. In the comparative analysis, cancer was linked to a 17.0 percentage-point decrease in the likelihood of functional independence (RD, −0.170; 95% CI, −0.223 to −0.116). Notably, the 95% PI (−0.248 to −0.091) was fully below zero, indicating that this association's direction persisted across the expected range of effects in future similar studies. Sensitivity analysis excluding studies at high risk of bias showed a similar pooled estimate, with 28.7% achieving mRS ≤2 at 90 days. The gap between successful reperfusion and subsequent disability, previously noted in thrombectomy studies involving patients with cancer [19,20], indicates that restoring large-vessel flow alone does not fully predict neurological recovery in this group. Overall, these findings suggest that restoring large-vessel patency alone may not be enough to counteract the systemic and oncological factors impacting stroke recovery [2,22].

Mortality followed a similar trend. The combined 90-day mortality rate for patients with cancer was 39.4% (95% CI, 31.6%-47.2%), and in-hospital mortality was 19.3% (95% CI, 14.0%-24.7%). Direct comparisons showed that cancer was linked to a 23.4 percentage-point increase in 90-day mortality risk (RD, 0.234; 95% CI, 0.168-0.300). The 95% PI for this outcome (0.111-0.357) was entirely above the null, indicating a consistent effect. Sensitivity analyses yielded similar 90-day mortality estimates for cancer patients, confirming that the findings were not solely influenced by high-bias studies. Past research and systematic reviews have also noted higher mortality rates among patients with malignancies, even after successful reperfusion [1,3,19,20,22]. This increased risk likely reflects factors such as advanced cancer, systemic illness, pre-stroke disability, stroke severity, treatment limitations, and other prognostic factors, rather than thrombectomy failure [1,2,22].

The hemorrhagic findings need to distinguish between symptomatic and overall intracranial bleeding. While sICH was rare and showed little variation between studies, the combined rate of any ICH was 30.2% (95% CI, 15.2%-45.3%), with significant differences among studies. Past systematic reviews have also noted variability in hemorrhagic outcomes after EVT in cancer patients, partly due to different definitions of hemorrhage and study populations [19,20]. Additionally, cancer-related issues in coagulation and vascular biology may influence hemorrhagic risk differently among patients [2,22]. Thus, the low sICH rate should not lead to the conclusion that broader postprocedural hemorrhagic events are absent.

Cancer-related stroke exhibits unique biological traits that may explain why reperfusion does not always lead to clinical recovery. Malignancy is linked with systemic hypercoagulability, platelet activation, inflammation, endothelial dysfunction, and other prothrombotic factors that can cause arterial thromboembolism [2,22]. These systemic effects may occur alongside frailty, anemia, infection, treatment complications, metastatic disease, and decreased physiological reserve. As a result, successful recanalization may not result in the same level of functional recovery or survival as in patients without cancer. Nevertheless, since most evidence is observational, these mechanisms are considered biologically plausible rather than definitively proven causal pathways. Factors such as cancer activity, metastatic load, ongoing treatments, pre-stroke functional status, comorbidities, and stroke severity likely influence these outcomes.

These results significantly impact EVT decision-making. The high recanalization rates and the lack of a noted increase in sICH compared to non-cancer controls indicate that simply having cancer should not automatically disqualify patients from EVT if they are otherwise suitable candidates [1,19,20]. However, the marked decrease in the likelihood of achieving functional independence and the higher 90-day mortality highlight that eligibility for the procedure and the overall potential benefit are not the same. Therefore, decisions should consider not only standard stroke treatment criteria but also factors such as cancer stage and activity, pre-stroke functional status, survival expectations, systemic disease extent, ongoing oncological treatments, and patient preferences. These findings support a personalized approach rather than routine exclusion or blanket treatment of patients with malignancies.

Several limitations must be recognized. First, the evidence solely comprised observational studies, with ROBINS-I assessments revealing moderate to serious bias risks in various cohorts. Key issues included residual confounding, incomplete adjustments for cancer severity and prestroke functional status, inconsistent definitions of malignancy, dependence on administrative coding in some large database studies, small cancer patient groups in certain studies, and the selective inclusion of patients deemed suitable for EVT. Second, there was notable clinical and methodological heterogeneity across several pooled outcomes, such as metastatic cancer, intravenous thrombolysis, any ICH, and mortality. Variations in cancer definition and activity, metastatic status, baseline stroke severity, EVT selection, treatment approaches, follow-up comprehensiveness, and outcome definitions likely drove this heterogeneity. Third, only a limited number of studies contributed to specific comparative outcomes, reducing accuracy and preventing reliable subgroup analyses or meta-regression. PIs should also be considered cautiously due to the small number of studies informing individual models. Fourth, oncological data, including cancer type, stage, activity, metastatic extent, treatment status, and expected survival, were inconsistently reported, hindering the identification of meaningful cancer subgroups. Lastly, no formal GRADE (Grading of Recommendations Assessment, Development, and Evaluation) assessment was conducted, and given the observational nature of the evidence, the pooled associations cannot confirm that cancer directly causes worse outcomes after EVT.

Overall, EVT in cancer patients showed a high success rate of recanalization and a low incidence of sICH, with no significant differences compared to non-cancer controls. However, these patients had notably lower 90-day functional independence and higher 90-day mortality. The persistence of these findings in sensitivity analyses, along with PIs favoring the same outcome, supports their consistency but does not eliminate the limitations of observational data. Future research should adopt standardized definitions for active and metastatic cancer, detail cancer stage and treatments, regularly consider prestroke functional status and stroke severity, and provide comprehensive EVT outcome data. These enhancements could better identify which cancer-associated stroke patients are most likely to benefit from thrombectomy.

## Conclusions

EVT in patients with cancer and AIS shows a high success rate in recanalization and a low risk of sICH. When compared to patients without cancer, there are no significant differences in recanalization success or sICH. However, patients with cancer tend to have lower functional independence at 90 days and higher mortality within that period. These results indicate that EVT can provide effective reperfusion in cancer patients, yet their overall outcomes are heavily influenced by factors beyond the procedure itself. Future research should include standardized cancer definitions, detailed oncological data, and consistent adjustments for prestroke condition and stroke severity to better identify those who will benefit most from EVT.

## References

[1] Navi BB, Kasner SE, Cushman M (2026). Classification and management of ischemic stroke in patients with active cancer: a scientific statement from the American Heart Association. Stroke.

[2] Dardiotis E, Aloizou AM, Markoula S (2019). Cancer-associated stroke: pathophysiology, detection and management (review). Int J Oncol.

[3] Zaorsky NG, Zhang Y, Tchelebi LT, Mackley HB, Chinchilli VM, Zacharia BE (2019). Stroke among cancer patients. Nat Commun.

[4] Verhoeven JI, Fan B, Broeders MJ, Driessen CM, Vaartjes IC, Klijn CJ, de Leeuw FE (2023). Association of stroke at young age with new cancer in the years after stroke among patients in the Netherlands. JAMA Netw Open.

[5] Ryan D, Bou Dargham T, Ikramuddin S, Shekhar S, Sengupta S, Feng W (2024). Epidemiology, pathophysiology, and management of cancer-associated ischemic stroke. Cancers (Basel).

[6] Page MJ, McKenzie JE, Bossuyt PM (2021). The PRISMA 2020 statement: an updated guideline for reporting systematic reviews. BMJ.

[7] van Swieten JC, Koudstaal PJ, Visser MC, Schouten HJ, van Gijn J (1988). Interobserver agreement for the assessment of handicap in stroke patients. Stroke.

[8] Sterne JA, Hernán MA, Reeves BC (2016). ROBINS-I: a tool for assessing risk of bias in non-randomised studies of interventions. BMJ.

[9] Verschoof MA, Groot AE, de Bruijn SF (2022). Clinical outcome after endovascular treatment in patients with active cancer and ischemic stroke: a MR CLEAN Registry substudy. Neurology.

[10] Lee EJ, Bae J, Jeong HB, Lee EJ, Jeong HY, Yoon BW (2021). Effectiveness of mechanical thrombectomy in cancer-related stroke and associated factors with unfavorable outcome. BMC Neurol.

[11] Włodarczyk E, Wrona P, Homa T (2024). Long-term outcomes of mechanical thrombectomy in acute ischaemic stroke patients with concomitant malignancy. Postepy Kardiol Interwencyjnej.

[12] Hsieh CY, Hsieh MT, Kim YS (2025). Temporal trends, outcomes, and predictors for endovascular thrombectomy in acute stroke patients with and without cancer. [IN PRESS]. J Formos Med Assoc.

[13] Shapiro SD, Vazquez S, Das A (2022). Investigating outcomes post-endovascular thrombectomy in acute stroke patients with cancer. Neurology.

[14] Ciolli L, Bigliardi G, Ferraro D (2021). Efficacy of mechanical thrombectomy in patients with ischemic stroke and cancer. J Clin Neurosci.

[15] Terazono S, Sakaeyama Y, Fuchinoue Y (2024). Usefulness of mechanical thrombectomy in patients with cancer who developed cerebral infarction. Neurol Med Chir (Tokyo).

[16] Merlino G, Smeralda C, Gigli GL (2021). Recanalisation theraphy for acute ischemic stroke in cancer patients. Sci Rep.

[17] Rinaldo L, Cloft HJ, Rangel Castilla L, Rabinstein AA, Brinjikji W (2019). Utilization rates of tissue plasminogen activator and mechanical thrombectomy in patients with acute stroke and underlying malignancy. J Neurointerv Surg.

[18] Fu CH, Chen CH, Lin YH (2025). High fibrin and platelet clot predicts stroke recurrence or mortality after thrombectomy in patients with active cancer. J Neurointerv Surg.

[19] Aloizou AM, Richter D, Charles James J, Lukas C, Gold R, Krogias C (2022). Mechanical thrombectomy for acute ischemic stroke in patients with malignancy: a systematic review. J Clin Med.

[20] Duan L, Fu Z, Zhao H (2022). Outcomes after endovascular thrombectomy for acute ischemic stroke patients with active cancer: a systematic review and meta-analysis. Front Neurol.

[21] Goyal M, Menon BK, van Zwam WH (2016). Endovascular thrombectomy after large-vessel ischaemic stroke: a meta-analysis of individual patient data from five randomised trials. Lancet.

[22] Navi BB, Iadecola C (2018). Ischemic stroke in cancer patients: a review of an underappreciated pathology. Ann Neurol.

